# Arginine depletion as a mechanism for the immune privilege of corneal allografts

**DOI:** 10.1002/eji.201141683

**Published:** 2011-07-29

**Authors:** Hongmei Fu, Adnan Khan, David Coe, Sarah Zaher, Jian-Guo Chai, Pascale Kropf, Ingrid Müller, Daniel F P Larkin, Andrew J T George

**Affiliations:** 1 Section of Immunobiology, Faculty of Medicine, Imperial College London, Hammersmith HospitalLondon, UK; 2 Moorfields Eye HospitalLondon, UK; 3 Institute of OphthalmologyLondon, UK; 4 Section of Immunology, Faculty of Medicine, Imperial College LondonLondon, UK

**Keywords:** Arginase, Cornea, Corneal graft, Immune privilege sites, Transplantation

## Abstract

The cornea is an immune privileged tissue. Since arginase has been found to modulate T-cell function by depleting arginine, we investigated the expression of arginase in the cornea and its possible role in immune privilege using a murine transplant model. We found that both the endothelium and epithelium of murine corneas express functional arginase I, capable of down-regulating T-cell proliferation in an in vitro culture system. The administration of the specific arginase inhibitor *N*-hydroxy-nor-l-Arg to recipient mice resulted in an accelerated rejection of allogeneic C57BL/6 (B6) corneal grafts. In contrast, in vivo blockade of arginase activity had no effect in altering the course of rejection of primary skin grafts that express little, if any, arginase. In addition, the inhibition of arginase did not alter systemic T-cell proliferation. These data show that arginase is functional in the cornea and contributes to the immune privilege of the eye, and that modulation of arginase contributes to graft survival.

## Introduction

The cornea is immune privileged, as evidenced by the high success rate of corneal grafts (typically 90% at one year 1) in the absence of systemic immunosuppression or HLA matching. There are several factors contributing to immune privilege 2, including (i) the absence of blood and lymphatic vessels in the graft bed 3, (ii) the expression of Fas ligand in corneal epithelial and endothelial cells 4, (iii) low expression levels of major histocompatibility complex class I and II molecules in corneal cells 5, 6, (iv) the atypical nature of the antigen-presenting cells within the cornea 7, (v) anterior chamber-associated immune deviation (ACAID) where systemic delayed-type hypersensitivity is down-regulated following exposure of the anterior chamber to alloantigens 8, and (vi) the presence of immunomodulatory cytokines such as transforming growth factor-β in aqueous humour in the anterior chamber of the eye 9.

l-arginine (l-Arg) is a semi-essential amino acid for adult mammals. Thus, while the body can synthesize some l-arginine, in some conditions, such as trauma and pregnancy, in which demand increases, it is necessary to supplement with dietary l-Arg 10. l-Arg is metabolized by two independent enzymatic pathways, involving the enzymes arginase and nitric oxide synthase (NOS) 11. Arginase hydrolyzes l-Arg into urea and l-ornithine, the latter being a precursor of polyamines (putrescine, spermidine and spermine) that are required for cell proliferation. NOS converts l-Arg into citruline and nitric oxide (NO), which are important in vascular homeostasis 11 and macrophage cytotoxicity 12.

The two identified arginase isoforms, arginase I and arginase II, differ in tissue distribution, subcellular localization and regulation 13. Arginase-mediated l-Arg depletion has been shown to down-regulate T-cell functions in various systems. Thus, tumour-associated myeloid cells express arginase I, and inhibition of the enzyme can slow tumour growth, suggesting that arginase I in the tumour microenvironment may be important for tumour escape 14, 15. T-cell functions are also suppressed by human granulocyte arginase 16. In human pregnancy, arginase activity is enhanced in both the peripheral blood and term placenta, resulting in T-cell hyporesponsiveness 17.

In this study, we investigated the hypothesis that arginase is involved in the maintenance of immune privilege in the eye. Our data show that both murine corneal endothelium and epithelium express functional arginase capable of down-regulating T-cell proliferation in an in vitro culture system. In a murine model of corneal allograft transplantation, inhibition of arginase in vivo accelerated corneal allograft rejection, but had no effect on the survival of transplanted donor skin that expresses little arginase. Therefore, arginase plays an important role in the maintenance of immune privilege in the eye by contributing to allograft acceptance.

## Results

### Functional arginase is expressed in murine corneas

**Figure 1:**
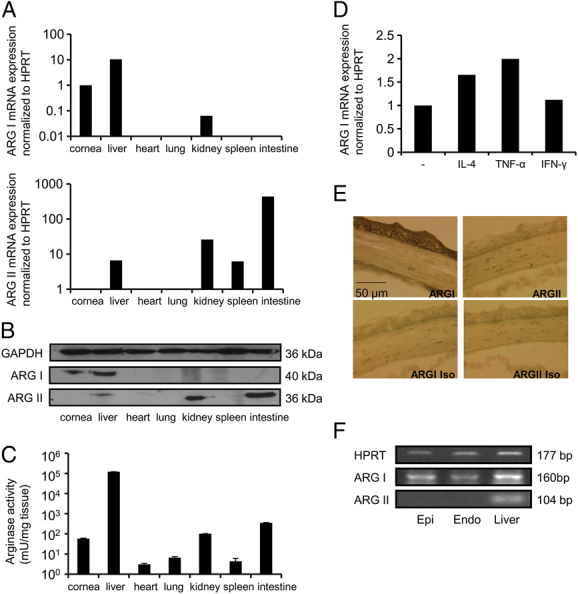
Expression of arginase I by murine cornea. (A) Real-time PCR analysis of arginase I (top) and arginase II (bottom) in murine cornea and other tissues. Total RNA was extracted from cornea and other tissues, and arginase mRNA was quantified by real-time PCR. Arginase mRNA levels were normalized to HPRT. (B) Western blotting for arginase protein. Tissues were homogenized and the supernatants were run on SDS-PAGE and probed for both arginase I and II. GAPDH was used as a loading control. (C) Arginase activity was measured in tissue extracts. Tissues were homogenized and the supernatants were taken to measure arginase activity by conversion of l-Arg into urea. Urea concentrations were assessed using a colorimetric assay, using the conversion of α-isonitrosopropiophenone; 1 unit of enzyme arginase activity catalyses the formation of 1 μmol urea/min. Data are represented as the mean+SD. (D) Effect of cytokine stimulation on arginase I mRNA expression in cornea. Arginase I mRNA levels were normalized to HPRT. (E) Immunohistochemical staining of the cornea with antibodies against Arginase I or II and isotype controls show expression in both the endothelium and epithelium. Magnification: 20×. (F) Epithelium and endothelium of cornea were separated surgically. Total RNA was extracted and arginase I mRNA expression was analysed by RT-PCR. Data are representative of three independent experiments, and in each group (A, C and D) triplicates were performed.

We examined the expression of arginase in whole murine corneas. BALB/c corneas were homogenized and tissue extracts were assessed for arginase expression at the mRNA and protein levels by real-time PCR and Western blotting, respectively. The expression of arginase was compared with that in liver, heart, lung, kidney, spleen and intestine. Arginase I mRNA was detectable in resting murine corneas (Fig. 1A). The level of arginase I in resting corneas was nearly a tenth of that in liver, which has the highest expression of arginase I. No expression of arginase I mRNA was found in heart, lung, spleen and intestine. The mRNA expression of arginase I was consistent with arginase I protein expression shown by Western blot using anti-arginase I antibody (Fig. 1B). In contrast, we were unable to detect arginase II mRNA or protein expression in the cornea. Strongest arginase II expression was seen in the intestine, followed by kidney and liver (Fig. 1A and B). To test whether the arginase present in the corneas was functional, we measured arginase enzymatic activity in murine corneas and compared it with other tissues. Arginase activity in unmodified resting murine corneas was easily detectable. It was lower than that in liver, intestine and kidney, but significantly higher than that in heart, lung and spleen (Fig. 1C).

To address, in part, the question of whether arginase function in vivo in the cornea might be altered during inflammation, corneas were cultured in the presence of murine cytokines IL-4, TNF-α and IFN-γ. These are reported to induce arginase expression in murine macrophages 18. A marginal increase in the mRNA expression of arginase I was seen in corneas stimulated with either IL-4 or TNF-α, but no change was seen with IFN-γ (Fig. 1D). No arginase II expression was seen under any conditions.

Immunohistochemistry was used to localize expression of arginase I and II in the cornea. Staining for arginase I but not arginase II was found in the endothelium and epithelium of the cornea (Fig. 1E). This was confirmed using RT-PCR analysis, which showed that arginase I mRNA was present in both the epithelium and endothelium of the murine cornea (Fig. 1F).

### Arginase in corneas has inhibitory effect on T-cell proliferation

**Figure 2:**
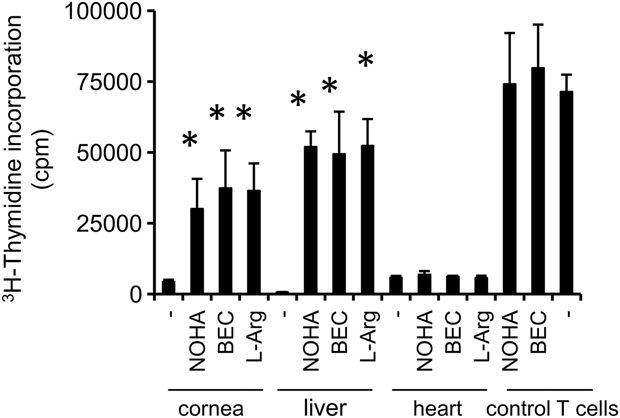
Effect of corneal arginase on T-cell proliferation. Tissues from cornea, heart and liver were homogenized and the supernatants (10 μL) were added to purified CD4^+^ T cells stimulated with anti-CD3 and -CD28 beads in the presence or absence of the arginase inhibitors, nor-NOHA, BEC, or 0.1 mM l-arginine in 96-well plates. T-cell proliferation was determined by ^3^H thymidine incorporation after 3 days (^*^p<0.05, Student's *t*-test). Data are representative of three independent experiments, and in each group triplicates were performed. Error bars indicate SD.

To examine the functional effect of corneal arginase, corneas were homogenized and the tissue extracts were added to purified CD4^+^ T cells stimulated with anti-CD3 and anti-CD28 beads in the presence or absence of competitive arginase inhibitors, *N*-hydroxy-nor-l-arginine (nor-NOHA), (S)-(2-Boronoethyl)-l-cysteine (BEC) or 0.1 mM l-arginine. ^3^H thymidine was added to T cells on day 4 and cells were harvested 16 h later. As shown in Fig. 2, addition of corneal extracts inhibited the proliferation of the T cells; this inhibition could be partially restored by addition of either nor-NOHA, BEC or l-arginine (*p*<0.05). These data indicate that arginase in corneal extracts is, in part, responsible for the observed inhibition of T-cell proliferation. A similar result was also seen with liver extracts. Addition of nor-NOHA or BEC had no effect on T-cell proliferation in the absence of tissue extracts. In contrast, addition of nor-NOHA had no effect on the inhibition of T-cell proliferation caused by lysates of heart tissue.

### Inhibition of arginase shortens corneal allograft survival

**Figure 3:**
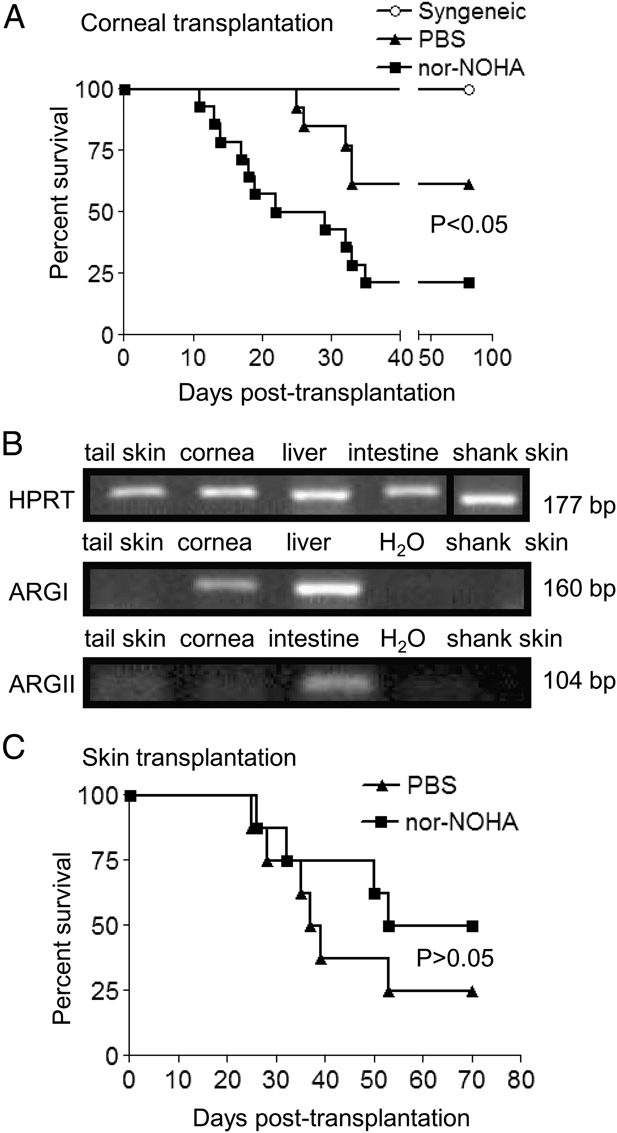
Inhibition of arginase shortens corneal allograft survival but not skin graft survival. (A) BALB/c (H2^d^) mice received unilateral C57BL/6 (H2^b^) donor corneal allografts and intraperitoneal injections of either nor-NOHA at 100 mg/kg/day (*n*=14) or PBS (*n*=13) from day 1 to day 21. Pooled results from two independent experiments are shown. Allograft survival was assessed by determining the opacity of the cornea using an objective scale, as described in 44. (B) RT-PCR analysis of arginase expression in donor tail skin and recipient shank skin. cDNA was prepared from tissues, and PCR was carried out using HPRT, arginase I and II primers and the product was run on a 1.5% agarose gel. Data are representative of three independent experiments. (C) B6 female mice received B6 male donor skin allografts and intraperitoneal injections of either nor-NOHA at 100 mg/kg/day (*n*=8) or PBS (*n*=8) from day 1 to day 21.

Having found that the cornea expresses arginase I, which inhibits in vitro T-cell proliferation, we then tested the possibility that functional arginase expressed by the cornea might prevent or delay corneal allograft rejection. As arginase knock-out mice do not live beyond 12 days 19, we used a competitive arginase inhibitor, nor-NOHA, in vivo. C57BL/6 (B6) strain mouse corneas were grafted as orthotopic transplants to BALB/c recipients randomized to receive daily intraperitoneal injections of either nor-NOHA at 100 mg/kg/day or control PBS from day 1 to day 21 post-transplantation. The rejection of B6 corneas in the nor-NOHA treatment group was accelerated compared with that of control group (*p*=0.014). Graft rejection onset ranged from day 11 to day 35, with 22% long-term survival. In contrast, control graft rejection ranged between days 25 and 33 with 61% long-term survival (Fig. 3A). Syngeneic grafts survived indefinitely.

### Inhibition of arginase has no effect on skin allograft survival

Inhibition of arginase might shorten corneal allograft survival either by local action on arginase activity within the transplant graft or by systemic modulation of the allograft response 20. To determine which is the case, we performed B6 male to female skin transplants. This donor–recipient combination was chosen because it has similar rejection kinetics as the B6 to BALB/c corneal transplantation model. RT-PCR analysis revealed that neither donor tail skin nor shank skin (site of the graft bed) express arginase I and minimal arginase II (Fig. 3B). Recipients were randomized to receive either nor-NOHA at 100 mg/kg/day or control PBS from day 1 to day 21 post-transplantation. Figure 3C shows that the rejection of skin grafts in the nor-NOHA treatment group is similar to that of the control group (*p*=0.6).

### Inhibition of arginase has no effect on systemic T-cell proliferation

**Figure 4:**
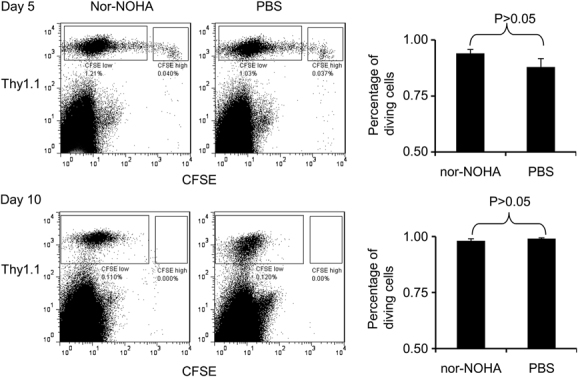
Inhibition of arginase has no effect on systemic T-cell proliferation. Splenocytes from Thy1.1^+^ Rag2^−/−^ Marilyn mice were stained with CFSE and adoptively transferred into B6 females (Thy1.2^+^) by i.v. injection. On the same day (day 0), all recipients were immunized with male splenocytes. From day 1 to day 7, B6 female mice received intraperitoneal injections of either nor-NOHA at 100 mg/kg/day or PBS. At days 5 and 10, three mice from each group were sacrificed and splenocytes were analysed by two-colour flow cytometry. Lymphocytes were gated on forward and side scatter and shown for expression of Thy1.1 (*y* axis) and CFSE (*x* axis). Three animals were used per time point for each group. Error bars indicate SD. Student's *t*-test was used to generate the *p*-values described.

To further investigate the effect of arginase inhibition on systemic T-cell response, we tested the ability of T cells to proliferate after administration of the arginase inhibitor, nor-NOHA. Splenocytes from Thy1.1^+^ Rag2^−/−^ Marilyn mice were stained with CFSE and adoptively transferred into B6 females (Thy1.2^+^) by i.v. injection. On the same day (day 0), all recipients were immunized with male splenocytes. From day 1 to day 7, the animals received either nor-NOHA at 100 mg/kg/day or PBS. At day 5 and day 10, three mice from each group were killed and splenocytes were stained for Thy1.1. Figure 4 shows a similar degree of proliferation of male-specific T cells in animals receiving nor-NOHA or PBS, suggesting that inhibition of arginase has little systemic effect on T-cell proliferation.

### The inhibition of arginase does not alter NO-dependent apoptosis of corneal endothelial cells

**Figure 5:**
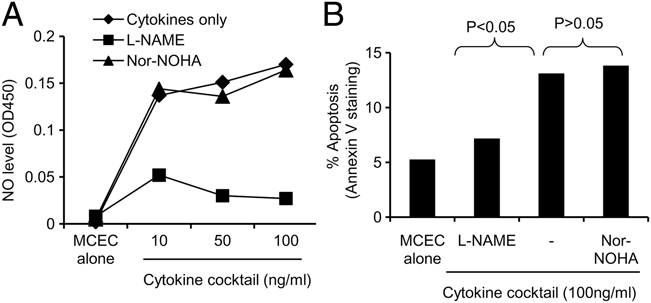
Nor-NOHA does not increase NO production by MCECs exposed to a cocktail of cytokines. MCECs were treated with different concentrations of the proinflammatory cytokines IL-1, TNF-α and IFN-γ (10–100 ng/mL) and cultured in the presence or absence of nor-NOHA (0.6 μM) or L-NAME (5 μM), for 48 h. (A) NO level in the supernatant of cultured cells using Griess reagent following conversion of nitrate into nitrite. (B) Analysis of cell apoptosis by Annexin-V staining. Data are representative of three independent experiments. Student's *t*-test was used to generate the *p*-values described.

Non-dividing corneal endothelial cells are critical for the maintenance of corneal transparency and thus are essential to graft survival 21. We have shown that the pro-inflammatory cytokines, IL-1, TNF-α and IFN-γ, induce apoptosis of corneal endothelium through an NO-dependent pathway 22. Since arginase and NOS share the same substrate 11, it is possible that the arginase inhibition would result in an increase in NO production through the NOS pathway, thus accelerating graft destruction. To investigate this, murine corneal endothelial cells (MCECs) were exposed to different concentrations of cytokines in the presence of either the arginase inhibitor nor-NOHA, or the NOS inhibitor L-NAME (5 μM). NO level in the supernatant of cultured cells was subsequently measured. As shown in Fig. 5A, the cytokines increased NO production by MCECs, as previously shown. L-NAME significantly reduced the production of NO; however, nor-NOHA did not alter the production of NO. Therefore, l-Arg was probably not limiting for NO production by NOS in this setting. As expected, considerable cell apoptosis was observed in MCECs stimulated with IL-1, TNF-α and IFN-γ. The NOS inhibitor L-NAME, but not the arginase inhibitor nor-NOHA, inhibited cytokine-induced apoptosis (Fig. 5B).

## Discussion

Corneal transplantation is highly successful without tissue matching and systemic immunosuppressive therapy due to the immune privilege of the eye, with around 90% of grafts surviving 1 year, though this drops to ∼75% at 5 years 23. Lack of lymphatic drainage 3, the atypical nature of the APCs within the cornea 7, limited MHC expression 5, 6, FasL expression 4, the tendency to induce anterior chamber-associated immune deviation 8 and immunosuppressive cytokines 24, 25 have all been associated with immune privilege. Here, we reveal for the first time that the murine cornea expresses functional arginase I that plays an important role in the maintenance of immune privilege of corneal allografts.

Arginase is expressed in various tissues and organs. Consistent with previous reports 26, we found that the murine liver expresses high levels of arginase I and low levels of arginase II. The kidney, intestine and spleen express only arginase II. Arginase, by virtue of its ability to deplete arginine, can be an effector mechanism of the immune system. Arginase expression by tumour and human term placenta have been found to suppress T-cell responses 14, 17. This is due to the l-Arg depletion by arginase. It has been demonstrated that l-Arg availability can modulate T-cell function. Arginase-mediated l-Arg depletion induces down-regulation of CD3ζ, the main signalling chain of TCR 27. Therefore, arginase has been suggested to play a role in local immune suppression.

This is the first report of functional arginase I expression in the cornea and several lines of evidence suggest that arginase may be an additional factor in the comparative immune privilege of this tissue. We found that both corneal endothelium and epithelium expressed arginase I. We also found that tissue extracts from murine corneas inhibited T-cell proliferation, which could be partly reversed by either nor-NOHA or BEC or by supplementation of the medium with l-arginine, thus demonstrating the presence of functional arginase in the cornea. When the competitive arginase inhibitor, nor-NOHA, was systemically administered, the rejection of corneal allografts was accelerated. Consistent with our finding, it has been reported that arginase I mRNA was present in murine cornea but arginase II mRNA was not detected. Expression of both arginase I and arginase II were elevated in the cornea at late stages of ocular herpes simplex virus type-1 infection 28.

Arginase can operate either locally or systemically to suppress the immune response 20. Therefore, the shortening of graft survival may be due to systemic, rather than local, effects. Consistent with another report 29, there is no arginase in the murine skin, although expression has been seen in human skin 30. However, in skin allografts, which do not express arginase I, there was no effect of nor-NOHA treatment. Furthermore, inhibition of arginase had no effect on the T-cell proliferation following systemic immunization. Therefore, it is likely that arginase is operating locally to down-regulate the rejection in the tissue, rather than affecting the priming or initial activation of the alloreactive T cells.

This is the first report of arginase function in transplantation immunology. While we have shown that arginase has a role in preventing corneal graft rejection, though not skin graft rejection, it is interesting to speculate that it may also have a role in other organ allografts. This is especially the case for the liver, which has high levels of arginase and allografts of which can undergo spontaneous acceptance in animal models 31, 32.

As arginase and NOS share l-arginine as their primary substrate 11, it is possible that inhibition of arginase might increase the production of NO. Inflammatory cytokines induce apoptosis of corneal endothelial cells in an NO-dependent manner 22, and it is conceivable that arginase inhibition might potentiate this effect. However, there was no difference in either NO levels generated by MCEC cells, or in their apoptosis, following stimulation by a cocktail of cytokines in the presence or absence of nor-NOHA. There are conflicting reports regarding the role of arginase in regulating NO synthesis. Some studies suggest that arginase activity regulates the availability of l-Arg as a substrate for NOS in endothelial cells 33, 34, while others show arginase activity has no effect on NO production in macrophages 35. These differences may reflect whether arginine is limiting in particular systems.

In higher organisms, the control of amino acid metabolism is a strategy for limiting the unwanted proliferation of cells, including T cells 36. l-Arg is not the only amino acid with effects on the immune system. Indoleamine 2,3-dioxygenase (IDO) is another enzyme metabolizing the essential amino acid l-tryptophan that can modulate the function of the immune system 37. IDO has been involved in the maintenance of immune privilege in the placenta 38. We previously found expression in murine cornea of functional IDO; overexpression of IDO in the donor corneas prolonged allograft survival. However, it is noteworthy that inhibition of IDO had no effect on corneal allograft survival 39. This report of arginase activity is another example of the ability of the eye to inhibit the proliferation of infiltrating T cells by enzyme-mediated amino acid starvation, contributing to the immune privilege of corneal allografts.

## Materials and methods

### Cell culture

SV-40-immortalized BALB/c corneal endothelial cells (MCECs) 40, a gift of Dr J. Y. Niederkorn, Dallas, TX, were maintained in Eagle's MEM supplemented with 1% (vol) penicillin and streptomycin and non-essential amino acids (Gibco-BRL, Paisley, UK) and 10% heat-inactivated FCS (Gibco-BRL) 22.

### RNA extraction and reverse transcription

Corneal or other tissues were ground using the beads and reagent supplied with the Fast RNA Pro Green Kit (QBiogene, Carlsbad, CA, USA). Corneal tissue was separated by dissection surgically into epithelium, and endothelium. RNA extraction and quantification was performed as previously described 21.

### Quantitative PCR

The PCR protocol was performed using an Applied Biosystems machine and the Power SYBR Green mastermix (Applied Biosystems, UK) with an initial denaturation step at 95°C for 5 min followed by 40 cycles of amplification (denaturation at 95°C for 5 s, annealing at 60°C for 10 s, elongation at 72°C for 13 s), and quantification at 81°C 39. Quantification of arginase I mRNA was carried out using the primers 5′-ACCTGGC CTTTGTTGATGTC-3′ (forward) and 5′-ACTGCCAGACTGTGGTCTCC-3′ (reverse) spanning 160 bp of the gene. Quantification of arginase II mRNA was carried out using the primers 5′-CTGCCATTCGAGAAGCTGG-3′ (forward) and 5′-GGGATCATCTTGTGG ACATTAG-3′ (reverse) (spanning 104 bp) 41. Quantification of hypoxanthine phosphoribosyl transferase (HPRT) mRNA was carried out using the primers 5′-ATGAT CCAGTCAACGGGGGAC-3′ (forward) and 5′-CCAGCAAGCTTGCAACCTTAACCA-3′ (reverse) (spanning 177 bp).

### Immunoperoxidase staining

Tissue sections (8 μm, embedded in OCT) were allowed to dry at room temperature for 2 h, treated with 3% H_2_O_2_ in methanol. Blocked with normal goat serum (1:25 with PBS) and then treated with 0.1% Triton X-100 in PBS. After three washes in PBS, sections were incubated with either rabbit anti-mouse arginase I or rabbit anti-mouse arginase II (Santa Cruz, USA) diluted 1:100 for 18 h at 4°C. After washing in PBS, sections were incubated with horseradish peroxidase- (HRP) conjugated goat anti-rabbit IgG (Dako, Denmark) diluted 1:50 for 1 h at room temperature. After washes with PBS, the chromogen 3,3′-diaminobenzidine tetrahydrochloride (DAB enhanced liquid substrate system; Sigma, D3939) was applied at 100–200 μL for 2–5 min. The sections were washed in tap water before haematoxylin counterstaining for 30 s.

### Western blotting

Corneal tissue was ground using the beads as described above. After centrifugation, the supernatant was separated on 10% SDS-PAGE under reducing conditions and transferred to nitrocellulose membrane using electrophoresis. Membranes were probed using either rabbit anti-mouse arginase I antibody or rabbit anti-mouse arginase II antibody, followed by HRP goat anti-rabbit IgG antibody. Blots were developed using ECL plus system (Amersham Pharmacia Biotech).

### Arginase activity

The enzymatic activity of arginase was measured as previously described 42.

### Cytokine treatment

Recombinant murine pro-inflammatory cytokines TNF-α, IL-1α and IFN-γ (PeproTech EC, London, UK) were added directly to cell cultures at final concentrations of between 10 and 100 ng/mL and cultured for 48 h.

### In vitro T-cell proliferation assays

Spleen cells from C57BL/6 mice were treated with a mixture of anti-CD45R/B220, anti-CD8 and anti-MHC class II supernatants (RA3-3A1, M5/114, 53.6.7 and 2.4G2) for 30 min. After antibody treatment, the cells were washed and incubated with goat anti-mouse IgG and goat anti-rat IgG-coated beads (Dynal, Bromborough, UK) for 30 min, followed by removal of bound cells with a magnet 39. Responder CD4^+^ T cells (1×10^5^ cells/well) were stimulated with beads coated with anti-mouse CD3 and CD28. Tissues were ground using the beads. After centrifugation, 10 μL of supernatant from the homogenized tissues was mixed with MnCl_2_ and added to the T cells in the presence or absence of 10 μM competitive arginase inhibitors L-2-Amino-(4-(2′-hydroxyguanidino)butyric Acid (nor-NOHA) or BEC, or 0.1 mM l-arginine in 96-well plates. Proliferation was measured by a 16-h pulse with ^3^H thymidine (10 μL, ∼5 μCi/mL, Amersham Pharmacia Biotech).

### Measurement of apoptosis by Annexin V staining

This has been done according to manufacture's protocol (BD Pharmingen™, 556421).

### Nitric oxide colorimetric assay

The NO level was measured using the Nitric Oxide Colorimetric Assay Kit (BioVision, K262-200) according to the manufacturer's protocol.

### In vivo T-cell proliferation assays

Spleen cells from Thy1.1^+^ Rag2^−/−^ Marilyn mice (TCR transgenic with specificity for HY/Dby peptide 43, provided by Dr. O. Lantz, Paris, France) were stained with CFSE (4 μM) and adoptively transferred into B6 females (Thy1.2^+^) by i.v. injection (2×10^6^ cells/mouse). On the same day (day 0), all recipients were immunized with male splenocytes. From day 1 to day 7, B6 female mice received intraperitoneal injections of either nor-NOHA at 100 mg/kg/day or PBS. At day 5 and day 10, three mice from each group were killed and splenocytes were stained for Thy1.1 marker. Cells were gated on Thy1.1 (eBioscience) positive population. Proliferation was measured by CFSE (Invitrogen) dilution.

### Corneal transplantation

Orthotopic corneal transplantation was performed using a modification of a technique described previously 44, 45. Briefly, donor C57BL/6 (H2^b^), (B6) corneas were excised using a 2.0-mm trephine (Duckworth & Kent, England). The right corneas of recipients were excised with Vannas scissors (Duckworth & Kent). B6 corneas were then applied to BALB/c corneal beds, secured with 8–9 continuous 11/0 nylon sutures. Sutures were removed on day 7, at which time any grafts showing technical failures were excluded from the study 44. The specific arginase inhibitor *N*-hydroxy-nor-Arg (Bachem, Germany) was injected intraperitoneally (i.p.) at 100 mg/kg/day for the treatment group (Control animals received the same volume of PBS). All animals were obtained from Harlan Olac (Bicester, UK) and treated in accordance with the ARVO Statement for the Use of Animals in Ophthalmic and Vision Research and United Kingdom Home Office Guidance.

### Skin transplantation

Skin grafting was conducted as described by Billingham and Medawar 46 using tail skin grafted onto the lateral thorax. Plasters were removed after 8 days and grafts were examined every 2 days until rejection (<10% of the original graft remains viable).

### Statistical analysis

Mean values with SD are shown where appropriate. Statistical significance was determined using Student's *t*-test. Differences in graft survival were analysed using log rank test and actuarial survival plotted according to the Kaplan–Meyer method. A value of *p*<0.05 was regarded as statistically significant.

## Abbreviations

BEC: (S)-(2-Boronoethyl)-L-cysteine
nor-NOHA: N-hydroxy-nor-L-Arg
MCEC: murine corneal endothelial cell
NOS: nitric oxide synthase
